# Speak like a Native English Speaker or Be Judged: A Scoping Review

**DOI:** 10.3390/ijerph182312754

**Published:** 2021-12-03

**Authors:** Kim Hua Tan, Michelle Elaine anak William Jospa, Nur-Ehsan Mohd-Said, Mohd Mahzan Awang

**Affiliations:** Faculty of Education, Universiti Kebangsaan Malaysia, Bangi 43600, Selangor, Malaysia; p105080@siswa.ukm.edu.my (M.E.a.W.J.); nurehsan@ukm.edu.my (N.-E.M.-S.); mahzan@ukm.edu.my (M.M.A.)

**Keywords:** accented speakers, cognitive, affective, and behavioral experiences, cyberbullying, pronunciation, scoping review

## Abstract

This review aims to examine the discrimination and prejudices toward the accent of non-native English speakers and cyberbullying as the ripple effect of these negative consequences. Following Arksey and O’Malley’s framework of conducting a scoping review, 60 studies from 2012 to 2021 were retrieved from the ERIC and Google Scholar databases. The studies were reviewed from two aspects: (1) psychological impact on speakers with a non-native English accent, (2) attitudes toward non-native English accents from the victim’s and perpetrator’s perspectives. The findings suggested that speaking with a non-native English accent drew negative cognitive, affective, and behavioral experiences. Biases toward non-native English accents were due to the general derogatory perception of an accent and the comprehensibility of speakers’ accent and pronunciation. “Accent acceptability” can be inculcated at all levels of education, not only through multicultural education but also through the concerted effort of policy makers and practitioners to seriously address this social issue. Accent awareness can dispel unwarranted and undesirable judgements of non-native English accent speakers. Future studies should be conducted on the effects of social and mental health experiences, particularly of non-native ESL and EFL teachers, given that this may be the only profession required to teach “live” during the pandemic and thus be subjected to public praise or ridicule.

## 1. Introduction

For decades, one of the most popular ideas in the linguistic field is that accent is part of a culture, which reflects a person’s identity. A listener’s view of the speaker’s accent can affect how he or she sees the person [1], which creates the first impression of an individual. The term accent has several meanings, but in speech it is an identifiable style of pronunciation that varies geographically or socioeconomically [1] and is one of the most noticeable features of speech [2]. Regional accents are influenced by geographical location, whereas social accents reflect speakers’ educational, socioeconomic, and ethnic backgrounds [2,3] as well as the phonetic variances caused by first language (L1) effect on the second language (L2) [2].

Recent theoretical developments suggest that speaking with an accent is related closely to the listeners’ verbal and nonverbal responses. Some learners evaluate their own accent and consider it superior to the accent of the others [4]. Spoken accents significantly impact and can influence listeners’ personal opinions [5]. Based on academic reports from the last 60 years, when social identity theory and ethnocentrism are viewed together, stating that higher ethnocentricity would cause listeners to have a negative perception of speakers with non-native accents is reasonable [6].

Proponents of English as a lingua franca movement as opposed to English as a foreign language teaching posit non-native speakers’ and all English varieties to be embraced in their own right and not be evaluated on the basis of a native speaker English (NSE) benchmark [7]. However, the central premise of this paper suggests otherwise, given that past studies have reported mixed findings of English as a second or foreign language (ESL/EFL) learners’ acceptance and perception of a variety of English that does not bear a close resemblance to NSE.

With the online mandatory teaching and learning arising from the COVID-19 pandemic, as well as a more active social media presence, more people are subject to scrutiny given that society members have easy access to online videos. Teachers and learners with non-native English accents are mocked, judged, and criticized incessantly [8,9]. This paper, therefore, seeks to investigate the impact on speakers with accented speech against speakers of native English accents by examining the domains and context in which it occurred, from past studies filtered from online databases. The domains involved are organized at the affective, behavioral, and cognitive levels [10,11]. Although the affective level describes the speakers’ personal emotions and behavioral level indicates measurable and/or observable changes among the speakers and listeners, the cognitive level involves the listeners’ perspectives of speakers with either native or non-native English accents. The studies reviewed also involved cyberbullying cases, which is behaviorally, affectively, and cognitively related and typically driven by speakers’ pronunciation or accents.

In this new era of technology, attitudes toward accents and pronunciation are largely manifested through cyberbullying, which refers to defamation, bullying, harassment; or discrimination; the disclosure of personal information; or the use of rude, vulgar, or disparaging comments to unsuspecting victims in the form of e-mail, instant messaging, or texting, blogs, internet postings, or social media [12]. In the context of accented speech, cyberbullies make those who speak with an unfavorable accent their target victims. Cyberbullying is increasingly becoming a social concern, as reflected by relevant studies in the present paper. It causes severe health issues for today’s adults and is a significant source of stress for adolescents and their households, while unraveling the ugly truth of cyberbullies’ easy access to more platforms and higher chances of demonstrating their aggression in the virtual world [13].

## 2. Materials and Methods

This review paper followed Arksey and O’Malley’s scoping review framework [14], together with the Joanna Briggs Institute System for the Unified Management, Assessment, and Review of Information (JBI SUMARI) to analyze papers on how accented speech and pronunciation among non-native speakers, and particularly those in the workplace, lead to cyberbullying. Four phases were involved in analyzing the literature (see Section 2.1, Section 2.2, Section 2.3 and Section 2.4)—from determining the research question to identifying relevant studies, choosing the articles, outlining the data, and presenting the results.

### 2.1. Scoping Review Research Question

The research question aimed at the initial state for defining the study’s framework and the topics spanned in the research question formulated the objective of the study [14]. The underlying research question is “what are the impacts of speaking with a non-native English accent?” The general concepts of “accent” facilitated the study so that a wide interpretation of accent is included, thus ensuring that the articles were centralized on accent and pronunciation in second language or foreign language speakers. The search criteria were broadened, and decisions on how to establish frameworks on the examined articles were made after the overall contents of the literature was assessed to acquire the scope of research in this field of study.

### 2.2. Relevant Studies

Relevant keyword searches based on the research question were retrieved from the ERIC website and Google Scholar electronic databases. The ERIC website database was chosen because it is the most widely used index of educational-related publications worldwide. As indicated in Table 1, articles from 2012 to 2021 were chosen; time range decisions are often necessary from a practical perspective in scoping literature reviews. The search terms used for this review were as follows:

i. “How does speaking with accent lead to cyberbullying”, with 87 results from ERIC database and 830 results from Google Scholar;
ii. “Speaking with accent” (20 results from ERIC website) (63.400 results from Google Scholar);
iii. “Attitudes towards speakers of non-native English accent” (1749 results from ERIC website) (16.100 results from Google Scholar);
iv. “Cyberbullying of teachers” (40 results from ERIC website) (16.700 results from Google Scholar).

### 2.3. Study Selection: Inclusion and Exclusion Criteria

**Figure 1:**
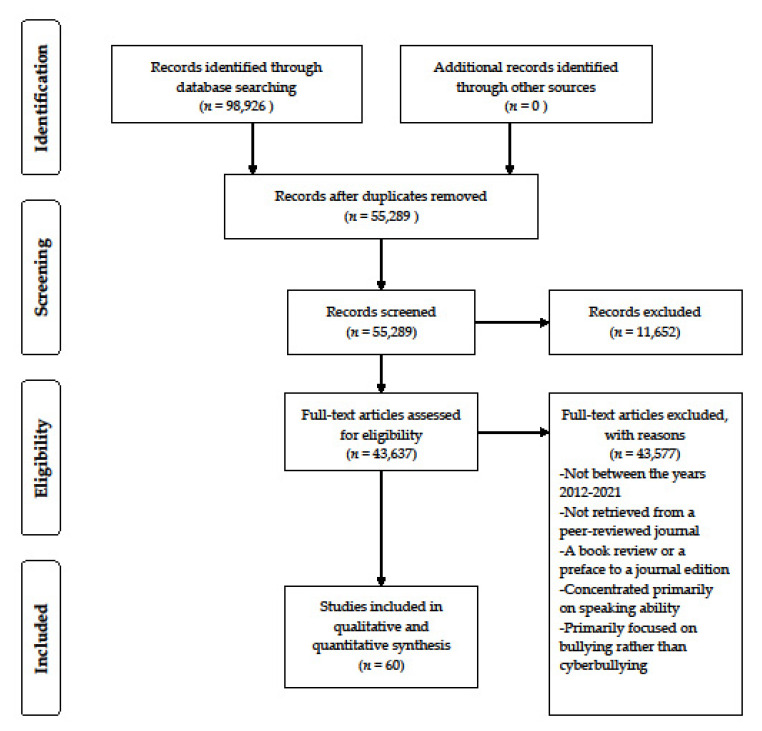
PRISMA flow diagram of the literature searches and study selection process.

As indicated in Figure 1, a total of 98.926 references were generated on the basis of the keyword searches. It comprises some duplicate articles discovered after numerous searches. After removal using Endnote, only 55.289 articles were left for further analysis. From the number, 11.652 articles were excluded, with 43.637 articles with full-text access remaining. After a pilot test was conducted, titles and abstracts were vetted by two or more independent reviewers and assessed on the basis of the review’s inclusion criteria. Their abstracts were evaluated for applicability to the present topic, theoretically relevant sources were downloaded, and their citation details were uploaded into the Joanna Briggs Institute System for the Unified Management, Assessment, and Review of Information (JBI SUMARI) to be further processed and validated. The full texts of the chosen citations were then examined and analyzed by two independent reviewers on the basis of the inclusion criteria to determine its subject to ensure relevance for the scoping review. The articles had to meet the following inclusion criteria to be considered for analysis:

After applying the criteria, 60 articles were selected to be included in the current review.

Adapted from: Moher, Liberati, Tetzlaff, Altman, and The PRISMA Group [15].

### 2.4. Charting the Data

After the selection process, each featured article was “charted,” or classified according to the review’s major topics and emerging themes. The papers in this scoping review were organized into three distinct domains, i.e., affective, behavior, and cognitive impact of speaking with a non-native English accent. These pertained to attitude to language with regard to pronunciation and accent. Therefore, a classification and definition by Lambert [8], which was later adopted by Gardner [9], were used. The demarcation of the three components was applied to indicate clearer implications on L2 learners’ and/or cyberbullying victims instead of grouping all these together.

## 3. Results

In total, 60 articles were examined to investigate the impacts of speaking with a non-native English accent, which were categorized into three aspects: (i) affective impact [16,17,18,19,20]; (ii) behavioral impact [21,22,23,24,25,26,27,28,29,30,31,32,33,34,35,36,37,38,39,40,41,42]; and (iii) cognitive impact [43,44,45,46,47,48,49,50,51,52,53,54,55,56,57,58,59,60,61,62,63,64,65,66,67,68,69,70,71,72,73,74,75]. Table 2 summarizes all 60 studies according to the year of publication, country, research design and sample, key findings, database the article was retrieved from, and the dominant domain of impact. The studies were predominantly from countries in Asia and Europe where English is spoken as a second or foreign language. These studies were from Asian countries, i.e., Malaysia, Taiwan, Pakistan, Vietnam, China, Japan, India, Philippines, Iran, Indonesia, Afghanistan, Saudi Arabia, Thailand, and Hong Kong. The rest of the studies were from European countries, i.e., Turkey, the Netherlands, Spain, France, Italy, Slovakia, and Canada. A total of 16 studies were from countries with a high number of immigrants historically—in the United States and the United Kingdom where English is the first language, English is also the working language of migrants in the workplace.

Affective impact indicates the consequences of speaking with a non-native accent on the speakers in relation to their emotions. Studies that reported affective impact described how the respondents’ or research participants’ feelings were affected. Five studies discussed this aspect of impact by non-native speakers from countries across the world, particularly in developing economies. All the studies were quantitative in nature (Table 2).

Compared with affective impact, more studies on behavioral impact were found, where 14 of them were quantitative in nature, 7 were qualitative, and 1 study was mixed method in nature (Table 2). Behavioral impact describes how speaking with a native or non-native accent leads to a situation that is measurable, usually through learning gains, linguistic achievement, or career development.

Studies on the non-native English accent reported mostly cognitive impact that describes how speaking with a native or non-native accent influences the perception of the listeners. Therefore, these studies were from the listeners’ perspectives. In total, 18 of them were of quantitative nature, whereas 9 employed mixed methods. Qualitative is the least employed approach with only 6 studies (Table 2), and there existed some empirical papers. The findings were derived from a verbal guise test to evaluate respondents’ attitude toward the three main ethnolects of Malaysian English: Malay, Chinese Malaysian, and Indian Malaysian’s English speech and to assess linguistic attitudes of participants regarding six types of accented lecturers’ speech. The studies also reported attitudes of students toward native and non-native English-speaking teachers and explored the impact of Native English-Speaking Teachers (NEST) and Non-Native English-Speaking Teachers (NNEST) on students’ English competency and their perceptions. One article examined the attitudes of English as a Foreign Language Learners on Different English Accents and NNEST in Pronunciation Teaching.

The psychological impact of speaking with a non-native English accent is tabulated according to affective, behavioral, and cognitive experiences of victims and perpetrators.

Table 2 delineates and summarizes these three experiences.

## 4. Conclusions

English is an international language used worldwide. However, spoken English varies due to the regional accent of certain places. Different types of attitudes exist owing to these variations, especially with non-native English speakers. Some fluent speakers and the natives of English can tolerate the variations of accents, whereas some show strong disapproval publicly, which is manifested online through cyberbullying. Less job prospects, bigotry, and under-appreciation of workers’ abilities are among the outcomes of the discernment of non-native English speakers’ inability to communicate [16,25].

Three impacts of speaking with a non-native English accent were identified on the basis of previous studies. The psychological impacts are classified into three main categories of experiences, namely, affective, cognitive, and behavioral experiences.

Regarding affective experiences, non-native speakers who speak English with an accent expressed negative feelings, such as annoyance, humiliation, and regret [17], from how they were treated by others. Some indicated that they felt excluded and undervalued at work [16], whereas others blatantly admitted that they were tired of having to speak in a foreign language and having to adhere to its linguistic rules [17].

These negative feelings are detrimental, given that they can lead to anxiety [18], which characterizes a mental and psychological problem. It affects all types of users, including the proficient ones. Some second language teachers in Slovakia still suffer from the issue as they expressed high levels of speaking anxiety and identified poor English pronunciation as the most significant obstacle in speaking [18]. Teaching experience does not reduce nervousness, and those who have been in service for quite long are still nervous about speaking due to their accent.

Studies that reported the behavioral impact were of mixed findings. Although the scarcity of subject specialists to teach English at school affected students’ speaking skills [23], learners have a higher chance of comprehension speech with non-native English teachers who spoke with an accent [21]. In listening lessons, utilizing materials with non-native English accents is more effective than native accent resources in facilitating learners’ listening comprehension [26]. Moreover, significant correlation exists between linguistic insecurities by NNEST and their learners’ writing and speaking performance, suggesting that accent does not impede learning at school [22].

Although speaking with a non-native accent does not matter as much in the educational setting given that it does not interfere with learning gain, ESL or EFL speakers with accents in other workplaces did not fare as well.. Those with a non-native English accent were discriminated against at work; they were trusted with more subordinate roles at work [23] and were less likely to be recommended for a middle management position [25]. As such, they had less opportunity to share input for administration and decision-making process compared with their native English speaker counterparts [23] who were assigned high posts at the workplace.

However, as far as cyberbullying victims are concerned, mockery on their pronunciation or accent significantly impacts in the educational setting and at work. On the one hand, victims displayed decreased academic achievement [27] and poorer self-esteem that led to higher anxiety and withdrawal from life experiences [30]. On the other hand, cyberbully victims at the workplace displayed negative interpersonal deviance [39] and increased stress level, which suggested their state of unhappiness at work [40]. Victims have also suffered from psychological trauma and humiliation when the mockery became public [41]. The condition is a serious issue of concern given that it results in reduced job satisfaction [42], which may eventually affect productivity if uncurbed.

Two attitudes were of prevalence from the literature for cognitive experience, with some listeners thinking highly of the native speaker accent, whereas others preferred the non-native English accent. Speaking with an accented speech elicits negative stigma [49], and listeners preferred the native English accent [43,54,57]. Respondents in the studies that were in favor of a native accent regarded the native speakers as the best models for pronunciation [42,48], especially when they were ESL/EFL learners with advanced proficiency [48]. Learners also considered the native speakers to be superior as a cultural repository [42] and more knowledgeable in grammar rules [34,43] and idioms [43]. Speakers who spoke with accurate words were always associated with native English language.

In addition to language proficiency, the major of study also influences the acceptance or non-acceptance of the non-native accent, given that law major undergraduates believe the native accent to be superior, whereas tourism major undergraduates were more accepting of native and non-native accents [50] and it is also reflective of cyberbullying, which was influenced by learners’ program of study and gender instead of ethnicity [58]. The audacity of cyberbullies arises from the state that they mocked their victims and managed to hide behind anonymity, eliciting lack of fear of punishment as people cannot easily recognize them [61].

Fundamentally, some respondents perceived the notion of intelligibility to be crucial for teachers and learners, rather than impeccable mastery of an idealized English variety [21]. These respondents, thus, were more receptive to the non-native accents [22,23] up to the extent that they rated the non-native accents more highly [32] as they perceived the version to have clearer and more comprehensible pronunciation for the ESL/EFL learners [40]. All non-native accents, such as Malay, Malaysian-Chinese, Malaysian-Indian, and Iraqi, were well received, but all native accents, such as American and British were assessed less favorably.

There appears to be positive and favorable sentiments regarding NNESTs by some learners [51], given that the teachers were considered one of them, and had also gone through the ordeal of learning a second language. Some non-native listeners can detect non-native speakers’ first language background on the basis of their English accents [55]. This includes ascribing certain characteristics to these speakers on the basis of preconceptions regarding the nationality to which they believe the speakers belongs. Hence, the attitudes that the listeners have toward the speakers are based on this notion. Attitudes may impact the perception of the comprehensibility of the speakers. The different variations of English are cherished as ESL/EFL called for a more active recognition and encouraging measures to increase awareness of the global expansion of English throughout the world, which has conceived many localized versions of the language [36].

These three experiences have left some non-native English speakers largely numb and disconnected and, eventually, the trauma dampened their spirits to communicate in English. Biases and prejudices toward these speakers with a non-native English accent should not be propagated, given that experts suggest that non-native accents are natural and, for the most part, an unavoidable companion of globalization and interlinguistic communication [76]. With the advent of Information and Communications Technology (ICT), the world has inevitably become a global village. People are communicating in English, despite having language barriers, given that English is the common language in most countries. Although some may use English with their regional accents, the message remains successfully delivered. The issue of fluency and accuracy may be regarded as secondary and periphery to successful communication.

Two common biases exist toward speakers with non-native English accents. The prejudices are related to the English competency of the speakers and comprehensibility of the speakers’ accent. Non-native speaker accents are less intelligible and comprehensible compared to native speakers [77]. Researchers have suggested that people may prefer to listen to English native speaker accents depending on contexts. In the educational context, some ESL/EFL learners prefer a non-native speaker accent, given that they comprehended non-native accents better than native accents. Unlike the educational context, a preference for native speaker accents exists in the workplace. Those who dislike the non-native English accents will eventually discriminate and turn to social media to express their dissatisfaction.

Despite efforts at multicultural education in the field of accents, a troubling level of non-native accent prejudice persists among speech language pathology students and practitioners [2]. Although these people have the privilege to be inducted into multicultural education, they remain prejudiced toward non-native accent speakers. This suggests that multicultural education alone is inadequate in creating awareness on the issue of respecting those who speak with a non-native English accent. More action must be taken to enhance the effectiveness of multicultural education in mitigating the prejudices and discrimination toward speakers with non-native English accents. The effect of social and mental health experiences, particularly among non-native ESL and EFL teachers, is perhaps crucial at this juncture. This is probably the only profession that is tasked to teach “live” during the pandemic and thus be exposed to public praise or ridicule. Accent awareness, accent tolerance, and what motivates accent acceptability should be inculcated in the workplace and taught at all levels of education. Until “accent acceptability” is highlighted by policy makers in any country, victims of online criticism will escalate because of the way they sound when they speak. Ultimately, the psychological impact is severe and probably non-reversible.

## Figures and Tables

**Table 1:** Summary of the Searches.

Search Terms	Limiters	Databases	Search Results
How does speaking with an accent lead to cyberbullying	Articles from 2012–2021	ERIC website electronic database	87
		Google Scholar electronic database	830
Speaking with an accent	Articles from 2012–2021	ERIC website electronic database	20
		Google Scholar electronic database	63.400
Attitudes toward speakers of non-native English accent	Articles from 2012–2021	ERIC website electronic database	1749
		Google Scholar electronic database	16.100
Cyberbullying of teachers	Articles from 2012–2021	ERIC website electronic database	40
		Google Scholar electronic database	16.700

**Table 2:** Summary of Information from the Selected Articles.

Author	Year	Location	Research Design	Sample	Main Findings	Database	Dominant Domain
[16]	2017	Italy	Quantitative	Numbers of sample are not stated.	Speaking with a non-native accent may cause speakers to (i) feel excluded and undervalued at work and (ii) adopt an avoidance strategy at work.	Google Scholar	Affective
[17]	2019	USA	Quantitative	*n* = 99	Non-native speakers reported stereotype threat, worry, weariness, status loss, unpleasant emotions, avoidance goal orientations, and avoidance. Furthermore, non-native speakers reported cognitive fatigue as a result of conversing in a foreign language.	Google Scholar	Affective
[18]	2018	Slovakia	Quantitative (Scale and Test)	*n* = 100	The positive association between age and pronunciation anxiety and negative relationship between age and pronunciation quality contradicts the common view that teaching experience duration is a role in reducing NNESTs’ nervousness.	ERIC	Affective
[19]	2016	Turkey	Quantitative (Questionnaire)	*n* = 160	The learners’ great tolerance for ambiguity in the classroom helps explain the perceived effectiveness of NESTs.	Google Scholar	Affective
[20]	2014	USA	Qualitative (Survey)	*n* = 613	College students reported that cyberbullying made them unhappy, angry, or agitated, and increased their stress, demonstrating that the psychological impact of cyberbullying does not fade as the victim ages.	Google Scholar	Affective
[21]	2016	USA	Quantitative (Comprehension tasks)	*n* = 115	Although greater background noise levels were often more detrimental to listeners with poor language skills, all listeners exhibited significant comprehension impairments with native speakers of English over RC-40. However, with Chinese speakers, the figure was lower.	Google Scholar	Behavioral
[22]	2017	Turkey	Quantitative (Questionnaires, semi-structured interviews, and proficiency tests)	Teachers *n* = 18 Learners*n* = 300	The linguistic insecurity of NNESTs, female and male, is not significantly connected to the learners’ writing and speaking scores.	ERIC	Behavioral
[23]	2014	UK	Qualitative (Interviews and Reflections)	*n* = 54	Generally, native English-speaking workers hold high positions and make critical decisions, whereas non-native English speakers hold more subordinate roles and have less input into organizational administration and decision making.	Google Scholar	Behavioral
[24]	2019	Pakistan	Qualitative (Interviews and observations)	*n* = 60	A scarcity of English language subject specialists affects the students’ speaking skill.	Google Scholar	Behavioral
[25]	2013	USA	Quantitative	*n* = 179	Non-native English speakers are less likely to be recommended for a position in middle management and have significantly lower chances of obtaining new-venture funding.	Google Scholar	Behavioral
[26]	2019	Iran	Quantitative (Quick Placement Test, Pearson Test ofEnglish General)	*n* = 60	Using non-native accent listening materials was more effective than using native-accent resources in improving EFL learners’ listening comprehension.	Google Scholar	Behavioral
[27]	2016	Pakistan	Quantitative (Survey)	*n* = 610	When socioeconomic status is not taken into account in the model, cyberbullying may considerably and negatively impact students’ academic achievement.	ERIC	Behavioral
[28]	2018	Spain	Quantitative (Questionnaire)	*n* = 1062	Cyberbullying crime was connected with cyberbullying victimization, bullying violence, moral disengagement from cyberbullying, social support, and display of enjoyment.	ERIC	Behavioral
[29]	2015	Canada	Qualitative (Survey)	*n* = 1001	Children who are cyberbullied are more likely to have unfavorable outcomes across all eight categories studied.	ERIC	Behavioral
[30]	2018	USA	Qualitative (Survey)	*n* = 187	Cyberbullying results in lower self-esteem, anxiety, and loss or withdrawal from social relationships and experiences.	ERIC	Behavioral
[31]	2016	Canada	Qualitative (Survey)	*n* = 145	With increased access to advanced technology and teenage fascination with it, cyberbullying is on the rise, and its harmful impacts on youth are being witnessed at school and at home.	Google Scholar	Behavioral
[32]	2018	Canada	Qualitative (Survey, interviews)	*n* = 192	Parental supervision of computer usage, students’ willingness to alert parents about cyberbullying, and how students and educators view the role of parents in cyberbullying prevention and promotion.	Google Scholar	Behavioral
[33]	2015	UK	Quantitative (Survey)	*n* = 158	Cyberbullying’s effects on trainee doctors.	Google Scholar	Behavioral
[34]	2017	UK	Quantitative (Questionnaire)	*n* = 331	The effects of cyberbullying and offline bullying.	Google Scholar	Behavioral
[35]	2021	UK	A mixed method (quantitative-qualitative) (Survey, interviews)	*n* = 144	The impact of workplace cyberbullying and whether it is more severe than traditional bullying.	Google Scholar	Behavioral
[36]	2020	Pakistan	Quantitative (Survey)	*n* = 329	Cyberbullying in the workplace causes negative consequences.	Google Scholar	Behavioral
[37]	2021	Thailand	Qualitative (Interviews)	*n* = 8	Several consequences that occur to victims during and after cyberbullying.	Google Scholar	Behavioral
[38]	2013	the Czech Republic	Quantitative (Survey)	*n* = 138	Cyberbullying seems to be a type of abusive student’s behavior directed toward their teachers.	Google Scholar	Behavioral
[39]	2020	Pakistan	Quantitative (Questionnaire)	*n* = 351	The findings, which are based on the conservation of resource theory and affective events theory, demonstrate that workplace cyberbullying affects interpersonal deviance.	Google Scholar	Behavioral
[40]	2020	Australia	Quantitative (Survey)	*n* = 254	The findings revealed that workplace cyberbullying increased perceived stress, which reflected worker’s unhappiness.	Google Scholar	Behavioral
[41]	2019	Kenya	Qualitative (Interviews)	Numbers of samples are not stated.	The impact of cyberbullying at work negatively influences productivity owing to psychological trauma, legal engagement, and embarrassment when it becomes public.	Google Scholar	Behavioral
[42]	2019	USA	Quantitative (Questionnaire)	*n* = 205	Being cyberbullied resulted in reduced social self-efficacy, and having lower social self-efficacy was related to reduced levels of work satisfaction.	Google Scholar	Behavioral
[43]	2013	USA	Quantitative (Speaking test)	*n* = 120	The impact of pronunciation factors on judgments of non-native speakers’ oral competency had a hierarchical priority.	Google Scholar	Cognitive
[44]	2014	Taiwan	Quantitative	Pre-service teachers *n* = 58	Same attitude to their roles as non-native English speakers concerning pronunciation development and teaching.	ERIC	Cognitive
[45]	2018	USA	Quantitative (Survey)	*n* = 40	Focusing on intelligibility rather than flawless mastery of an idealized variation of English would benefit English learners and practitioners.	Google Scholar	Cognitive
[46]	2020	Flanders and the UK	Quantitative (Survey)	*n* = 232	Non-native English accents are accepted.	Google Scholar	Cognitive
[47]	2016	China	Quantitative (Pre- and post-tests, questionnaires)	*n* = 30	The participants’ comprehensibility and accentedness enhanced significantly. The majority of the participants would rather have a native speaking teacher than a non-native speaking teacher as their oral English teacher.	Google Scholar	Cognitive
[48]	2018	USA	Quantitative (Survey)	Numbers of sample are not stated.	Strong connections exist between the view of intelligibility and the perception of non-native speakers’ personal attributes.	Google Scholar	Cognitive
[49]	2012	USA	Quantitative	*n* = 20	These findings support previous studies, indicating that speakers’ accents significantly impact how others perceive them.	Google Scholar	Cognitive
[50]	2015	Malaysia	Mixed methods (quantitative-qualitative) (Survey)	*n* = 372	A strong positive correlation exists between each ethnic group’s attitude toward the Malaysian English variety spoken and the intelligibility of that specific variation, which significantly influences listeners’ opinion of the speaker’s social attractiveness.	ERIC	Cognitive
[51]	2014	Malaysia	Quantitative (Verbal-guise technique)	*n* = 120	The students displayed an in-group accent bias, which meant that they rated non-native lecturers’ accents more highly.	ERIC	Cognitive
[52]	2018	The Netherlands	Quantitative (Questionnaire)	*n* = 183	Non-native English listeners’ assessment of attitude was influenced by degree of accentedness in English, educational background, and language sensitivity.	Google Scholar	Cognitive
[53]	2014	Malaysia	Quantitative (Questionnaire)	*n* = 36	In terms of correctness, acceptability, pleasantness, and familiarity, respondents consistently evaluated native speaker accents higher than non-native speaker accents.	Google Scholar	Cognitive
[54]	2013	Philippines	Mixed methods (quantitative-qualitative) (Survey)	*n* = 120 Korean participants	Koreans are particularly susceptible to Philippine English vowel and consonant variations. When given the option of having a Philippine English speaker as their English teacher, the majority of the sample gave a negative response.	Google Scholar	Cognitive
[55]	2018	India and Iran	Quantitative (Questionnaire)	*n* = 260	Alongside supporting and honoring different variations of English, recognizing and encouraging measures to improve teacher and learner awareness of the global expansion of English are critical.	ERIC	Cognitive
[56]	2015	Indonesia	Qualitative (Interviews)	*n* = 204	Generally, neither native English speakers nor non-native English speakers are favored by the perceived attributes of an ideal English instructor established in this study.	Google Scholar	Cognitive
[57]	2019	Taiwan	Qualitative (Two open-ended questions)	*n* = 20	Generally, the participants preferred NESTs over NNESTs.	ERIC	Cognitive
[58]	2019	Indonesia	Qualitative (Case study)	*n* = 10	Despite having a very strong accent, speeches with clear and accurate pronunciation are considered highly accepted and totally understood.	Google Scholar	Cognitive
[59]	2012	Vietnam	Quantitative (Survey, questionnaire)	*n* = 50	Advanced English respondents chose native speaker of English because they regarded native speaker as the best model to learn pronunciation.	ERIC	Cognitive
[60]	2014	Vietnam and Japan	Quantitative (Questionnaire)	*n* = 100	Students perceived NESTs as representations of proper language use and pronunciation, as well as cultural information repositories.	Google Scholar	Cognitive
[61]	2014	Afghanistan and UK	Mixed methods(Questionnaire, structured interviews)	*n* = 90	Students highlighted the following strengths of their NESTs in questionnaires and structured interviews: teaching ability, grammaticality and idiomaticity, usage of the standard English language accent, and competency in managing spontaneous replies in the classroom.	Google Scholar	Cognitive
[62]	2018	Hong Kong	Mixed methods (quantitative-qualitative) (A listening task, survey, interview)	*n* = 21	The findings suggest that all participants favored native English as the paradigm of teaching and learning.	Google Scholar	Cognitive
[63]	2013	Malaysia	Quantitative (Questionnaire)	*n* = 34	Using a native accent as a model for pronunciation acquisition is a more practical alternative.	Google Scholar	Cognitive
[64]	2020	Malaysia	Qualitative (Essay writing)	*n* = 30	Most students had a poor opinion of NNESTs, particularly when it came to teaching grammar and speaking skills. The NESTs, despite being evaluated favorably at the start of the study, had a rise in negative responses from students.	ERIC	Cognitive
[65]	2017	Turkey	Mixed methods (quantitative-qualitative) (Questionnaire)	*n* = 42	Specific ideologies, such as standard English, native-speakerism, and authenticity impact many students’ normative judgments of good English.	ERIC	Cognitive
[66]	2012	Saudi Arabia	Mixed methods (quantitative-qualitative) (Questionnaire, interviews)	*n* = 169	As the respondents progress to higher levels, NESTs become more popular.	ERIC	Cognitive
[67]	2017	Spain	Qualitative (Textbooks analysis)	*n* = 14	Law students tend to appreciate native accents more than non-native accents, although tourism students typically accept native and non-native accents.	Google Scholar	Cognitive
[68]	2016	Taiwan	Quantitative (Questionnaire, interview)	*n* = 200	Taiwanese students’ sentiments regarding their non-native speaking English teachers are positive and favorable.	ERIC	Cognitive
[69]	2015	France	Quantitative (Survey)	*n* = 78	The majority of respondents stated that they preferred native English speakers as educators.	Google Scholar	Cognitive
[70]	2020	Thailand	Mixed methods (quantitative-qualitative) (Classroom observations, interviews)	*n* = 252	NESTs score better agreeability with teachers’ teaching abilities, English abilities, and the establishment of an interesting learning environment.	ERIC	Cognitive
[71]	2020	Turkey	Mixed methods (quantitative-qualitative) (Survey, interviews)	*n* = 169	Many participants agreed that proper pronunciation is essential in communication, and if a pronunciation is intelligible, it can be considered as good.	ERIC	Cognitive
[72]	2021	Netherlands	Quantitative (Questionnaire)	*n* = 522	Dutch and foreign non-native listeners rated moderately non-native accented lecturers adversely compared with lecturers with slight or native accents.	Google Scholar	Cognitive
[73]	2020	Malaysia	Quantitative (Survey)	*n* = 400	Gender and program of study are more predictive of undergraduates’ cyberbullying experiences than race.	ERIC	Cognitive
[74]	2016	USA	Qualitative (Survey)	*n* = 936	The major reasons for cyberbullying are anonymity, the cyberbully not realizing the real-life consequences of their actions, and a lack of fear toward punishment.	Google Scholar	Cognitive
[75]	2021	USA	Quantitative (Survey)	*n* = 823	Adult and peer assistance decreased the social and psychological suffering caused by cyberbullying.	Google Scholar	Cognitive

## Data Availability

Not applicable.
